# *Journal of Translational Medicine* advances in Translational Genomics and Genetics Era

**DOI:** 10.1186/s12967-019-1874-7

**Published:** 2019-04-25

**Authors:** Wei Liu

**Affiliations:** AbbVie Biotherapeutics, Redwood city, California USA

**Keywords:** Genomics and Genetics, Next generation sequencing, Bioinformatics, Noninvasive prenatal screening/testing, Single cell sequencing, Liquid biopsy and circulating tumor DNA

## Abstract

We are delighted to announce the launch of a new section in the *Journal of Translational Medicine*, ‘Translational Genomics and Genetics’. Central to translational medicine trend is the ability to sequence, annotate and interpret the genomic variants in a given patient to guide precision diagnosis and personalized treatment. This new section intends to promote the translation of emerging genomic technologies into clinical applications. The coverage extends to basic, translational and clinical studies related to human genomics and genetics. The manuscripts in following categories are welcome, but not limited to: Application of next generation sequencing in clinical diagnosis, including but not limited to rare genetic disease, complex inherited disease, prenatal/perinatal screening, oncology, organ transplantation and pathogen identification; Novel bioinformatics approaches in human genomics; Single cell sequencing, liquid biopsy and other emerging genomic assays in molecular genetics, pharmacogenomics, oncology and immuno-oncology; Integration of genomics with other “omics” technology in genetic medicine.

In this Editorial we announce a new section in the Journal of Translational Medicine: Translational Genomics and Genetics. This section provides a translational medicine forum for the publication of research on novel genomics assays and bioinformatics approaches that leads to better clinical diagnosis and treatment. The core objective of this new section is to welcome novel genomic assays and multi-omics biomarkers to delineate the underlying mechanism of human rare and complex genetic disease, cancer and infection disease.

The advent of next-generation sequencing (NGS) has changed the landscape of human genomics research and genetic diagnosis. Whole Exome Sequencing (WES), along with Whole Genome Sequencing (WGS) and capture based disease focused panel sequencing, had made rapid transition into routine rare disease clinical diagnosis and management. NGS has revolutionized molecular genetic diagnostic practices, enabled unprecedented and robust discovery of novel disease-associated genes and genetic variants. Although the individual diseases are rare, collectively together there were approximately 25–50 million Americans affected by rare genetic diseases. Therefore, rare diseases pose an enormous burden for the affected families and healthcare system. NGS enabled early diagnosis and optimal management had made some rare diseases compatible with a good quality of life [1–3].

Lo et al. first reported the presence of fetal DNA in maternal blood (circulating cell free fetal DNA, ccffDNA) back in 1997. Due to the low percentage of fetal DNA compared to maternal derived cell free DNA, despite intensive effort to use varies PCR and real time PCR technologies for non-invasive prenatal diagnosis, the application of ccffDNA was limited to fetus sex determination and Rhesus D genotyping detection etc [4, 5]. The emerging of NGS technology finally enabled non-invasive prenatal testing (NIPT) of common chromosomal aneuploidies (trisomiy 21, 18, and 13) and sex chromosome abnormalities. The commercial based on either shallow whole genome sequencing of ccffDNA or targeted sequencing to identify fetal specific single nucleotide variants. Beyond whole chromosome aneuploidies, the sequencing technology advancement as well as the drop of sequencing cost even enabled non-invasive prenatal screening of multiple Mendelian monogenic disorders [6].

The development of NGS and small or big molecule therapies that target cancerous genomic aberrations has accelerated the introduction of genomic assays into clinical cancer testing. Beyond the identification of targetable mutations, NGS based tumor mutation burden, neoantigen identification and inflammation status related gene expression signatures, have supported the development of immuno-oncology therapeutics such as checkpoint inhibitors as well as personalized cancer vaccines [7, 8]. Liquid biopsy is a technology that interrogates circulating tumor DNA and/or exosomes DNA genomic aberrations in the plasma of cancer patient via deep sequencing. Liquid biopsy opens a new avenue for early cancer screening, prediction of response or resistance to given therapies, monitoring minimum residue diseases and tracking treatment induced secondary resistance etc. in a non-invasive way [7].

As the costs of NGS technologies continue to drop, Direct-to-Consumer (DTC) genetic testing based on NGS becomes more accessible and commercially viable. The commercial DTC whole-genome or -exome sequencing enabled the generation of personal genetic data that bypasses the involvement of clinician. The big concern in medical community is that current interpretation of genomic data is limited to our understanding of medical knowledge. Therefore, there are a lot of ambiguities in what the DCT companies informs their clients about the meaning of specific sequence variation. In response of this concern, FDA and other regulatory agencies are intensively discussing new measures to strengthen the regulation of DTC genetic testing and avoid over-reach interpretive claims concerning medical risk associated with specific genetic variant [9].

To reflect the rapid transformation enabled by NGS technology in molecular and clinical genetic testing including rare disease diagnosis, pre-implantation, prenatal and perinatal genetic screening, cancer testing and liquid biopsy, pharmacogenomics as well as direct-to-consumer genetic screening, this new section of *Journal of Translational Medicine* intends to be an open and multidisciplinary forum of scientific exchange and interaction for better precision diagnosis and personalized treatment in the post-NGS era. This is also in line with the broader-spectrum objective of the Journal of Translational Medicine of optimizing the communication between basic and clinical science.
